# Caffeine Attenuates Electroacupuncture Effect on Pressure Pain Threshold and Tolerance in Healthy Individuals: A Randomized Controlled Trial

**DOI:** 10.3389/fneur.2022.859624

**Published:** 2022-07-07

**Authors:** Kun Liu, Xiang Cui, Mujun Zhi, Meng Zhang, Ting Zhao, Xinyan Gao, Bing Zhu

**Affiliations:** Department of Physiology, Institute Acupuncture and Moxibustion, China Academy of Chinese Medical Sciences, Beijing, China

**Keywords:** coffee, electroacupuncture, quantitative sensory testing, sensory perception, conditioned pain modulation

## Abstract

**Introduction:**

The effect of caffeine on acupuncture analgesia in humans is unclear. This study aimed to investigate whether caffeine-containing beverage intake influences the effect of electroacupuncture (EA) on static quantitative sensory testing (QST) and dynamic QST in healthy subjects.

**Methods:**

A total of 40 healthy subjects were enrolled and randomly assigned to receive coffee containing moderate doses of caffeine (coffee group) or non-caffeinated juice (juice group) for 4 weeks. The primary outcome measures were the pressure pain threshold (PPT), pressure pain tolerance (PPTo), and heat pain threshold (HPT) as static QST parameters. Numerical rating scales (NRS) of heat stimulus and nociceptive flexor reflex (RIII reflex), as parameters of dynamic QST, were also examined. EA stimulation with tolerance intensity was performed at ST36 (Zusanli)-GB34 (Yanglingquan) points at weeks 0, 2, and 4. PPT, PPTo, and HPT were detected pre- and post- EA. The NRS scores were examined pre-, during, and post-EA, and 1 min after EA was completed. The RIII reflex was examined pre- and 1–5 min post-EA.

**Results:**

At week 0, both groups showed increased PPT and PPTo and decreased NRS scores of heat stimuli and RIII reflex after EA, but HPT was not affected. After 4 weeks, the effects of EA on PPT and PPTo were attenuated in the coffee group compared to the juice group, whereas the effect of EA on the NRS scores and RIII reflex were not influenced. There was no significant difference found at week 2 for these indications. EA also did not affect the HPT in both groups at week 4.

**Conclusion:**

Moderate caffeine intake reduced the effects of EA on PPT and PPTo in healthy subjects.

## Introduction

Acupuncture, which has been practiced in China for thousands of years, is widely used to alleviate both acute (1) and chronic pain (2, 3) and is currently practiced in 160 countries and regions worldwide. Studies on the physiological, anatomical, and neurochemical mechanism of the analgesic effect of acupuncture have shown that the same and adjacent segmental acupuncture analgesia is attributed to the spinal gate control theory, in which the painful site activates Aβ fibers to suppress Aδ or C fiber activation (4). The supraspinal structures involved in the endogenous descending inhibitory system in the CNS contribute to heterosegmental acupuncture analgesia. This diffuse noxious inhibitory control (DNIC) system exerts its effect through the activation of C-fibers from high-intensity stimulation that in turn induces an analgesic effect (5). Many signal molecules are involved in acupuncture analgesia, and these are mostly known as opioid peptides (μ-, δ-, κ-receptors). Human and rodent studies have revealed that different frequencies of electroacupuncture (EA) increase the levels of different opioid peptides. For example, EA stimulation at 2 Hz increased enkephalins and endorphins in the CSF content, whereas 100 Hz stimulation increases the release of dynorphins in parabrachial nuclei (6, 7).

In addition, adenosine is reported to suppress acute and chronic pain in both preclinical animal models and human subjects. Activation of the A1 adenosine receptor (A_1_AR) plays an anti-nociceptive effect in spinal, supraspinal, and peripheral neurons, as well as glial cells (8–11). Other subtypes of adenosine receptors involved in pain modulation include the A2a adenosine receptor (A_2a_AR) and A2b adenosine receptor (A_2b_AR), which exhibit pro-nociceptive role in the periphery and spinal anti-nociceptive effects (8). Meanwhile, the role of the A3 adenosine receptor (A_3_AR) in pain conditions is complicated, but the use of A_3_AR agonists produces beneficial effects in neuropathic pain (12, 13).

Caffeine is a non-selective adenosine receptor antagonist. Coffee is a widely consumed caffeine-containing beverage that functions as a stimulatory agent in the central nervous system to increase alertness and decrease fatigue. The estimated daily consumption of caffeine is 168–410 mg/day in Western countries, while people in Eastern countries consume only 14 mg/day of caffeine. High, moderate, and low caffeine consumption is defined as consumption of 200–1,000, 100–200, and <100 mg/day, respectively (14, 15). Clinically, low doses of caffeine have an adjuvant analgesic effect that can inhibit A_2a_AR and A_2b_AR (16). Meanwhile, moderate to high doses of caffeine can block A_1_AR and reduce the anti-nociceptive effects of analgesics (17). Some clinic or preclinic studies have reported the anti-nociceptive effect of adenosine on acupuncture analgesia (18). Peripheral and central A_1_AR activations play a role in the anti-nociceptive effects of acupuncture (19, 20).

An animal study has shown that oral or local administration of caffeine during acupuncture eliminated acupuncture analgesia in acute and chronic animal pain models (21). In addition, a study of human subjects found that traditional acupuncture at Zusanli increased local interstitial adenosine concentration (22). In contrast, other studies have also shown that caffeine does not attenuate experimentally induced ischemic pain, and daily caffeine consumption does not influence acupuncture analgesia in healthy subjects (23, 24). Therefore, the effect of caffeine on pain in animal and human subjects is still unclear, and no systematic investigation has been conducted on the influence of caffeine on acupuncture analgesia in humans.

Quantitative sensory testing (QST), also called psychophysical testing, involves static and dynamic QSTs. Static QST further comprises the pressure pain threshold (PPT), pressure pain tolerance (PPTo), and heat pain threshold (HPT), while dynamic QST comprises conditioned pain modulation (CPM). CPM is used to experimentally assess endogenous pain inhibition and can be assessed using different methods. PPT, PPTo, and HPT are well-suited to provide confirmatory results on the mechanisms underlying acupuncture (25).

This study aimed to investigate whether caffeine-containing beverage intake influence the effect of electroacupuncture (EA) on static QST and dynamic QST in healthy subjects. Based on previous research on the biological effects of caffeine, we hypothesized that moderate doses of caffeine intake may inhibit the analgesic effects of acupuncture. Static and dynamic QST and CPM were assessed. The nociceptive flexion reflex of the lower limb (RIII reflex, a spinally mediated withdrawal reflex as a physiological correlate of spinal nociception processing) was also assessed to reflect the descending brain-to-spinal cord modulation of spinal nociception.

## Materials and Methods

### Study Design and Participants

**Figure 1:**
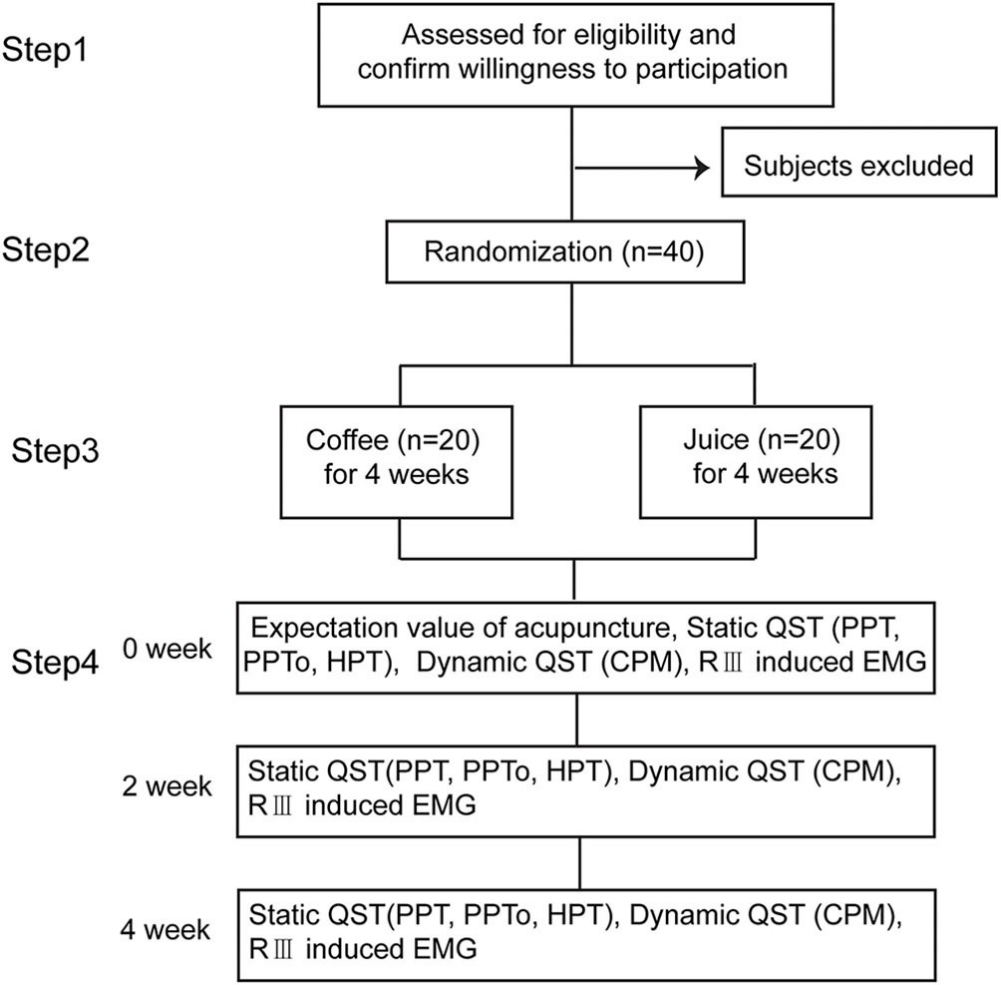
Study design. The participants are randomized in a 1:1 ratio to either the juice or coffee group. The static QST, dynamic QST, and RIII induced EMG are measured before and after acupuncture and at 2 and 4 weeks after intervention in each group.

This interventional randomized parallel controlled trial was conducted at the Electrophysiological Laboratory of the Institute of Acupuncture and Moxibustion, China Academy of Chinese Medical Sciences (IAM-CACMS). We enrolled healthy subjects from families, friends, colleagues who were familiar with acupuncture therapy, and postgraduate students at the Beijing University of Chinese Medicine and the China Academy of Chinese Medical Sciences. The inclusion criteria were aged between 20 and 40 years and no daily high caffeine-containing food or beverage (coffee, tea, chocolate, energy drinks, etc.) consumption habits. The exclusion criteria were (1) lactation or gestation; (2) a pacemaker; (3) lack of oral communication skills; (4) acute or chronic pain conditions or diabetes mellitus; (5) a history of chronic internal, dermatological, neurological, or psychiatric diseases; and (6) recent sleep deprivation or unusual physical exercise. Any kind of analgesic, anti-depressant, or cough suppressant was not allowed during the study period. All prospective subjects were asked for a medical history and underwent a comprehensive brief physical examination. If the subject met the study criteria, the nature of the study was explained and informed consent was obtained from the subject by our interviewer as in Step 1, Figure 1. Each participant scored the expectation value of acupuncture.

This study was approved by the China Ethics Committee of Registering Clinical Trials (No. ChiECRCT-20150031) and conducted according to the Declaration of Helsinki. The details of the study are reported in the Acupuncture-Moxibustion Clinical Trial Registry (registration number: AMCTR-IOR-18000164; http://www.acmctr.org/index.aspx) = ChiCTR1800015994 (http://www.chictr.org.cn) in the Chinese Clinical Trial Registry.

### Sample Size

The target sample size was calculated based on the ability to detect a PPT or PPTo difference between groups after a 4-week coffee intake or juice intake, given an expected PPT of 368.50 ± 119.14 kPa before acupuncture, 516.46 ± 207.41 kPa after acupuncture, 80% power, and 5% two-tailed significance level (26). The sample size was calculated using the STATA 14.0 software (Stata Corp., USA) for 10% attrition. In total, 40 participants were recruited (20 per group). The subject inclusion flowchart is shown in Figure 1.

### Randomization and Masking

The participants were randomly assigned in a 1:1 ratio to receive coffee or juice according to a randomization sequence performed by an independent researcher not involved in the examination or data analysis. The randomization sequence was generated using a computerized, random number generator with the Microsoft Office Excel 2010 software package. Allocation concealment was ensured by opaque envelopes labeled by the study participant number conveyed to an interviewer.

The research coordinator, who was not involved in data collection and data analysis, provided the participants with the beverage they would drink daily for 4 weeks. This coordinator also supervised all the participants to drink coffee or juice during the trial. The subjects were instructed to prepare the coffee or juice powder into 200 ml of warm water and answer drinking forms after each drink to record the time of drinking. The daily videos or video calls of drinking through their cellphones were sent only to the coordinator. During the video call, the participants would show the drink to the coordinator and report the time and date before they drank it off. The examiner, data collection staff and data analysts in charge of examining the participant, and recording data or data analysis were blinded to group allocation during the study period. The interviewer, coordinator, and the data collection staff and analysts were instructed not to exchange information during the entire period of the trial. Allocation concealment was not performed until the completion of the study. The tests and measurements were conducted in the same shielded room of the Electrophysiological Laboratory under similar conditions (quiet atmosphere, room temperature: 20–25°C, and humidity 40–60%).

### Intervention

The daily diet of the participants was maintained during the research except for the intake of coffee or juice. Subjects in the coffee group drank 2 bars of instant coffee (NESCAFE 1+2 original), with 50–60 mg caffeine/bar. Therefore, the daily consumption of caffeine in the coffee group was ~100–120 mg per person as a moderate dose (27). Meanwhile, subjects in the juice group were asked to drink non-caffeinated juice powder (TANG, Kraft Foods (Mondelēz International), 15 g per person daily. The examination was conducted from 1:00 pm to 5:00 pm. The participants were asked to finish drinking at least 1 bar of the coffee or juice assigned before arriving at the laboratory on the examination day.

**Figure 2:**
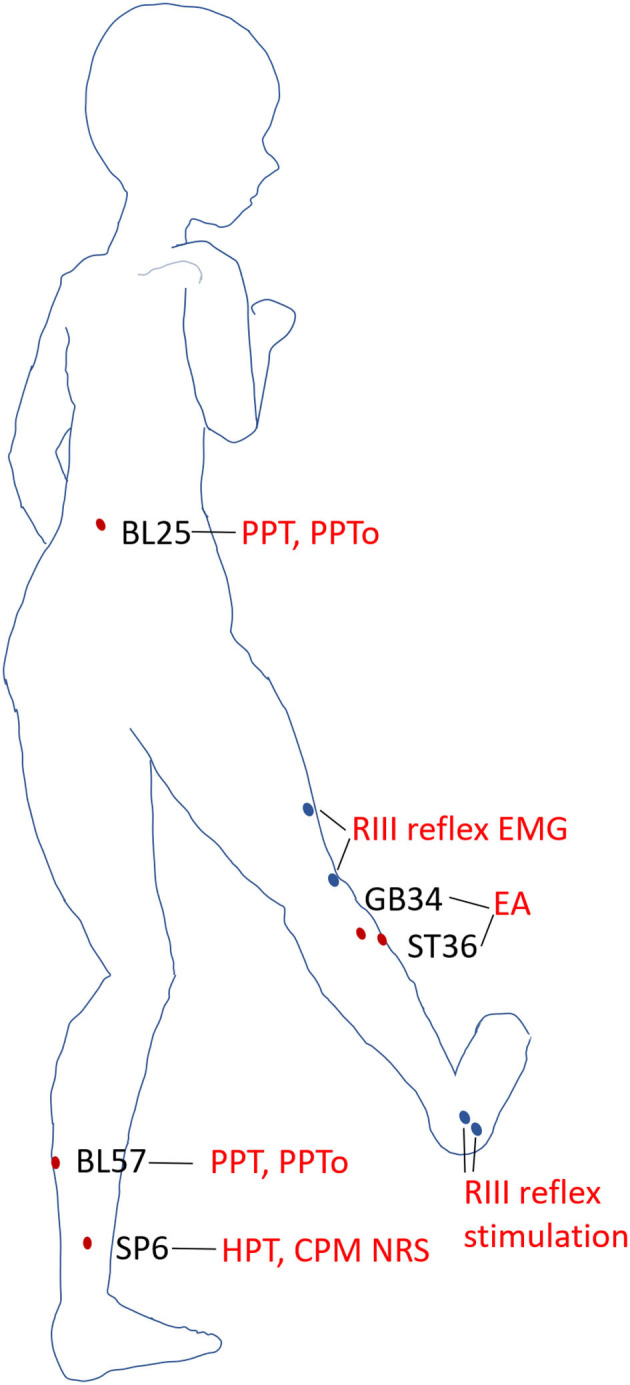
Acupoints and areas of QSTs. Schematic diagram of the acupoints and areas tested in static and dynamic QSTs. Electroacupuncture (EA) was performed on ST36 and GB34. PPT and PPTo were examined at BL25 and BL57 in the prone position. HPT and NRS scores of heat stimulus were measured at SP6 when the subjects reclined relaxedly. The areas of RIII reflex stimulation and recording were blue dots in the diagram.

The acupoints for EA were ST36 (Zusanli) located on the anterior aspect of the leg, on the line connecting ST35 with ST41; 3 B-cun inferior to ST35; and GB34 (Yanglingquan) located on the fibular aspect of the leg, anterior and distal to the head of the fibula. The acupoints for PPT and PPTo measurement were the left point of BL25 (Dachangshu, in the lumbar region, at the same level as the inferior border of the spinous process of the fourth lumbar vertebra (L4), 1.5 B-cun lateral to the posterior median line) and BL57 (Chengshan, on the posterior aspect of the leg, at the connecting point of the calcaneal tendon with the two muscle bellies of the gastrocnemius muscle). The acupoint for HPT measurement was SP6 (Sanyinjiao) located on the tibial aspect of the leg, posterior to the medial border of the tibia, and 3 B-cun superior to the prominence of the medial malleolus (28). The schematic diagram of the acupoints stimulated and areas of QSTs tested were shown in Figure 2.

EA was performed by an acupuncturist certified in Chinese medicine, using disposable acupuncture needles (0.25 × 40 mm, Huatuo, Suzhou Medical Co. Ltd., Jiangsu, China). Two needles were inserted perpendicularly into the acupoints at the left ST36 and GB34 ~25 mm in depth and then rotated clockwise and anticlockwise to induce a needle sensation. The EA current intensity induced a strong sensation within the subject's tolerance (15 Hz, 0.4 ms) to the HANS-200A analgesia apparatus (Nanjing Gensun Medical Technology Co. Ltd., Nanjing, China).

**Figure 3:**
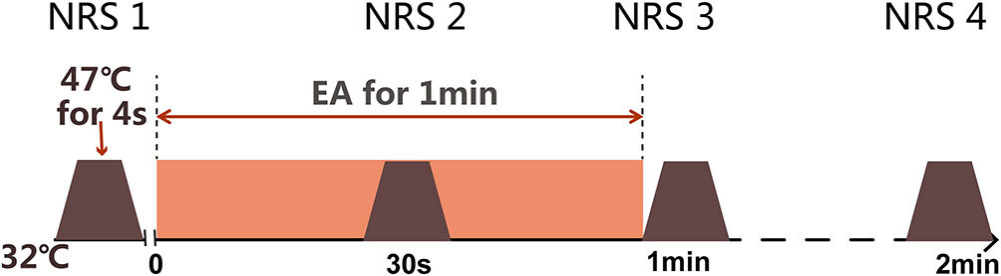
Measurement of dynamic QST (CPM NRS). Numerical rating scale (NRS) scores are obtained under heat stimulus of 47°C at SP6 for 4 s before electroacupuncture (EA), during EA, immediately after EA, and 1 min after EA.

**Figure 4:**
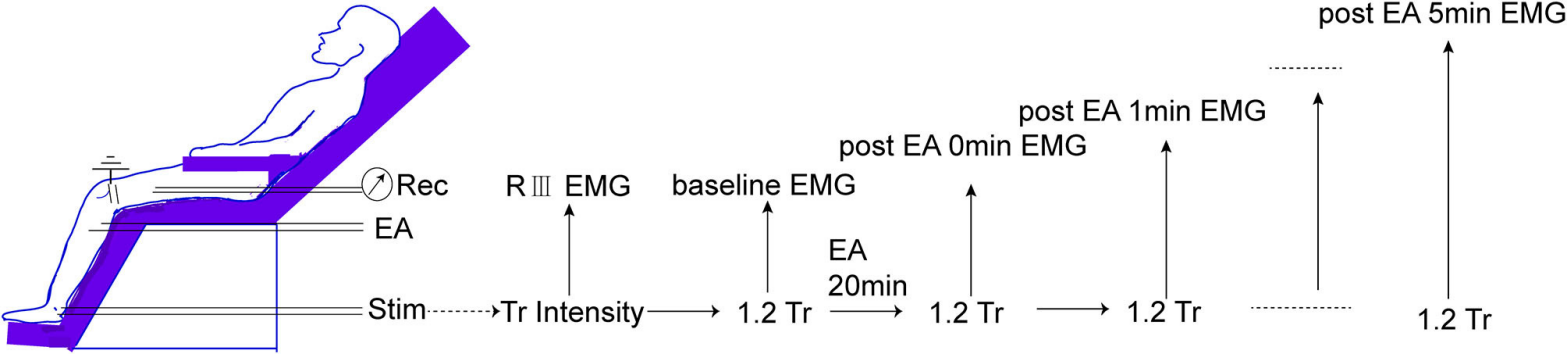
Measurement of RIII reflex-induced EMG. The threshold of RIII reflex (Tr) is determined according to high intensities of peroneal nerve stimulation. Then, 1.2 Tr peroneal nerve stimulus is conducted every 1 min before and after 0–5 min of EA.

Before any beverage intake interventions, the numerical rating score (NRS) of heat stimulus was measured on SP6 at 0 weeks before, during (30 s), and 0 min and 1 min after EA on ST36-GB34 (Figure 3). PPT, PPTo, HPT, and RIII (Figure 4) were assessed before and after 20 min EA. PPT, PPTo, HPT, NRS, and RIII reflexes were examined at weeks 0, 2, and 4 (step 4 in Figure 1). The sequence of testing was that PPT, PPTo, HPT, and RIII reflex before 20 min EA. Then, NRS before, during, and 0 and 1 min after EA were examined. After 20 min EA, RIII reflex, PPT, PPTo, and HPT were reexamined. PPT, PPTo, and HPT were examined in the prone position, CPM NRS and RIII reflex were done when the subjects were reclined relaxedly. The examinations are described in detail below. The examiner was trained for 1 week to ensure correct and precise assessments according to the German Research Network on Neuropathic Pain (29).

### Outcome Measures

The primary outcome measures were PPT, PPTo, and HPT. The subjects were placed in a prone position in a quiet room, and the static QST process was explained before the assessment.

#### PPT and PPTo

PPT and PPTo were measured using a pressure gauge device (FPIX25, Wagner Instruments, USA) with a contact probe (rubber tip) of 1 cm^2^ area, a measuring range of 100 N/cm^2^ (1,000 kPa), and a sensitivity of 0.1 N. The probe was perpendicularly pressed at a constant speed on BL25 or BL57. The PPT was read on the digital panel of the device if the subject verbally reported feeling pain, whereas PPTo was obtained if the subject reported intolerable pain. The same procedure was repeated three times for each subject.

#### HPT

Heat pain thresholds were detected using TSA 2001-II (Medoc, Ramat Yishai, Israel) with a thermode probe and a contact area of 16 ^*^ 16 mm^2^. The probe was fixed to the left SP6 with a gentle and complete contact with the skin securely. The baseline temperature was set at 32°C with a heating rate of 1°C/s, and cut-off at 50°C. HPT was recorded using the computer software WinTSA 5.19 with the subject clicking a mouse when he or she felt heat pain. This measurement was repeated three times per subject. The computer screen was not visible to the subjects.

#### CPM NRS

NRS as dynamic QST in this study was measured using TSA 2001-II. Before the test, the subjects were informed that they will be exposed to a short heat stimulus (47°C) on left SP6 through the contact of thermode. This was the testing stimuli. EA at ST36 for 1 min as described in the intervention part was the conditioning stimuli. The base temperature was 32°C and was increased to 47°C at a rate of 2°C/s, which lasted for 4 s before decreasing to 32°C again (Figure 3). The subjects were then asked to give an NRS score from 0 to 10, according to the following criteria: 0 for no pain, 2.5 for uncomfortable feeling, 5 for pain at threshold, 7.5 for moderate pain, and 10 for unbearable pain (30, 31).

#### RIII Reflex

The RIII reflex was assessed to test the effects of caffeine intake on pain threshold and EA benefit. The subjects were reclined relaxedly as depicted in Figure 4. All electrode sites were shaved, cleaned, and abraded with NuPrep gel (Weaver and Company, USA). A reference (common ground) electrode was attached to the lateral condyle of the femurs. RIII reflex-induced electromyography (EMG) of the biceps femoris muscle of the left leg, 10 cm superior to the popliteal fossa, was recorded by surface electrodes [3 M Medical Devices Materials and Manufacturing (Shanghai) Co. Ltd, China].

The EMG signal was amplified using a bioelectric amplifier (ML135, AD Instruments, Australia) with high-cut 1 kHz and low-cut 10 Hz and recorded using Powerlab 8/30 acquisition system (ML870, AD Instruments, Australia), and online or offline analysis was processed with LabChart software (version 7.3.7) with a digital filter set between 25 and 450 Hz for noise screen with a sampling rate of 4 K/s.

The retromalleolar pathway of the sural nerve was stimulated using a stimulator (DS5—Isolated Bipolar Constant Current Stimulator, Digitimer Ltd., UK) with two Ag-AgCl surface electrodes placed 2 cm apart onto the shaved and degreased skin (eight pulses in 20 ms, and each pulse for a duration of 1 ms; for each time, four series of the above pulses were given at 0.2 Hz). The individual threshold of the RIII reflex (Tr) was the minimum stimulus intensity that evoked the EMG of the biceps femoris muscle. Baseline EMG for each subject was determined by a 1.2 Tr electric stimulus. The subjects then received EA stimulation as described in the intervention session. The EMG induced by 1.2 Tr was recorded every 1 min continuously at 0, 1, 2, 3, 4, and 5 min after EA. No change in the outcomes was made after the trial commenced.

### Statistical Analysis

Data are presented as the mean and standard deviation. Data were first analyzed to determine whether they accorded to a normal distribution (Kolmogorov-Smirnov test: *P* > 0.05). All the baseline and change scores were found to be normally distributed in the sample. Baseline QST data between the two groups were analyzed by an independent *t*-test. To observe the change in analgesia efficacy of acupuncture, all data were calculated as the difference between post-EA and pre-EA. The difference in PPT, PPTo, and HPT was calculated by subtracting the baseline values from those after EA. The difference in CPM NRS scores and RIII reflex EMG integral was defined as the value during (30 s)/after (1–5 min) EA minus the value before EA. A general linear model was used to analyze group-by-time interactions. For PPT, PPTo, and HPT, the group was a fixed effect and the week was a random effect. For CPM and RIII reflex, the group was also a fixed effect and the time point was a random effect. The difference at the same week/time point between the groups was analyzed using the *t*-test. Data were analyzed using the Statistical Package of Social Science (SPSS, IBM SPSS Statistics for Windows, version 20.0; IBM Corp, USA) and SAS 9.3 (SAS Institute, USA). *P* < 0.05 was considered statistically significant.

## Results

### Participant Characteristics and Stimulation Parameters

All 40 subjects completed the intervention session, and no subject dropped out. There was no significant difference in the mean age and sex between the coffee and the juice group. The acupuncture expectancy scores were also not significantly different (Supplementary Table 1). No adverse events were reported. Sensory data obtained by QST and statistical differences are shown in Tables 1, 2 and Supplementary Tables 2, 3.

**Table 1:** Static QST change.

	**Least square mean (SD)** ^**†**^		**Mean score difference (95% CI)^**‡**^**
	**Coffee**	**Juice**	
**PPT (KPa)**			
BL25 Baseline	43.77 (19.84)	42.48 (19.84)	1.29 (−54.35, 56.93)
2 wk	27.45 (19.84)	40.88 (19.34)	−13.43 (−68.36, 41.51)
4 wk	−46.40 (20.39)	72.23 (20.98)	−118.64 (−176.64, −60.65)***
BL57 Baseline	39.09 (17.96)	31.72 (18.42)	73.72 (−43.62, 58.37)
2 wk	34.20 (17.96)	22.91 (18.93)	11.29 (−40.43, 63.01)
4 wk	−30.80 (17.96)	51.10 (19.48)	−81.91 (−134.42, 29.40)***
**PPTo (KPa)**			
BL25 Baseline	55.43 (21.47)	16.46 (24.01)	36.47 (−23.71, 96.65)
2 wk	18.97 (21.47)	21.72 (22.03)	−27.53 (−63.72, 58.22)
4 wk	−35.01 (21.47)	37.10 (21.47)	−72.11 (−132.30, −11.94)
BL57 Baseline	41.60 (22.25)	2.60 (22.83)	39.00 (−24.15, 102.16)*
2 wk	20.17 (22.25)	35.12 (22.25)	−14.95 (−77.29, 47.39)
4 wk	−12.53 (22.25)	62.93 (22.25)	−75.47 (−137.80, −13.13)*
**HPT (** **°** **C)**			
Baseline	0.58 (0.37)	0.30 (0.37)	0.28 (−0.75, 1.32)
2 wk	−0.05 (0.37)	0.30 (0.37)	−0.36 (−1.39, 0.68)
4 wk	−0.11 (0.38)	−0.28 (0.43)	−0.18 (−0.95, 1.30)

^†^*General linear model for repeated measures with group as fixed effect, week as random effect*.

*^‡^t-test.*

*Significant difference between Coffee and Juice group, *P <0.05, ***P <0.001.

**Table 2:** Statistical analysis: *P*-values from general liner models for repeated measure.

	**Time**	**Group**	**Time × group**
**PPT**
BL25	0.317	0.009	0.007
BL57	0.374	0.164	0.021
**PPTo**
BL25	0.289	0.507	0.043
BL57	0.969	0.336	0.042
**HPT**	0.317	0.538	0.777
**CPM NRS**
0 wk	0.018	0.126	0.795
2 wk	0.024	0.387	0.976
4 wk	0.284	0.809	0.808
**RIII reflex EMG integral**
0 wk	0.330	0.054	0.832
2 wk	0.992	0.001	0.935
4 wk	0.935	0.001	0.781

### Effect of Coffee on PPT and PPTo

The baseline PPT and PPTo values (kPa) for BL25 and BL57 showed no difference between the coffee and juice groups at 0 weeks initially (Supplementary Table 2).

For the changes in PPT values (difference between post- and pre-EA), there was a significant time-by-group interaction in the PPT of BL25 (*P* = 0.007) and BL57 (*P* = 0.021) (Table 2). The change scores of PPT at week 4 were significantly different between the coffee and juice groups (*P* < 0.001, Table 1). After coffee consumption for 4 weeks, the effect of EA on PPT was attenuated.

As shown in Table 1, there was a significant time-by-group interaction in the PPTo of BL25 (*P* = 0.043) and BL57 (*P* = 0.042) (Table 2). After 4 weeks of beverages intake, the changes in PPTo (difference between post- and pre-EA) at BL25 and BL57 in the coffee group were significantly lower than those in the juice group (*P* < 0.05, Table 1). After coffee consumption for 4 weeks, the effect of EA on PPT was attenuated.

### Effect of Coffee on Thermal Pain

There was no significant difference in baseline HPT between the coffee and juice groups (*P* = 0.730). Time-by-group interactions showed that coffee and juice intake did not affect the changes in HPT values before and after EA (*P* > 0.05, Table 2).

### Effect of Coffee on CPM

As shown in Supplementary Table 2, the baseline average NRS scores in the coffee and juice groups had no significant difference between the two groups before EA (*P* = 0.775). In both groups, pain levels measured by CPM NRS were significantly lower during (T1) and after (T2 and T3) EA than at baseline (Supplementary Table 3). This indicated the CPM effect of EA at 47°C heat stimulus. However, there was no significant between-group difference in the CPM effect at weeks 0, 2, and 4. There was also no time-by-group point interaction (*P* > 0.05, Table 2).

### Effect of Coffee on RIII Reflex

**Figure 5:**
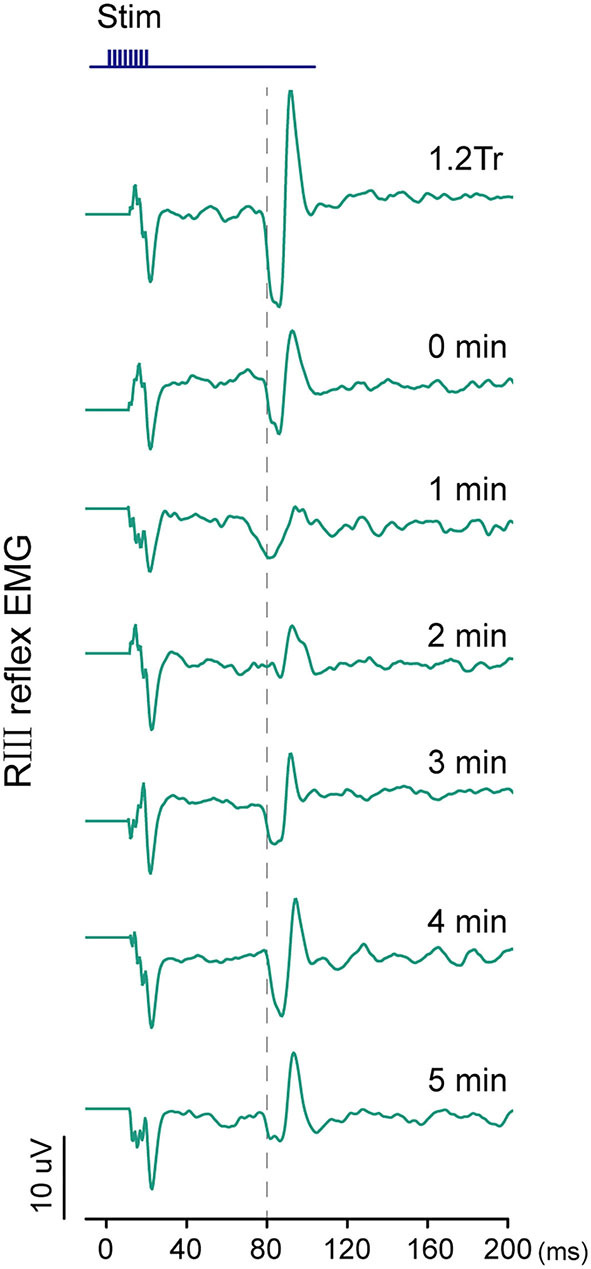
Examples of the changes in RIII reflex EMG at baseline by 1.2 Tr, immediately after (0 min), and 1–5 min after electroacupuncture (EA) application in the juice groups at week 0.

An example of an RIII reflex was shown in Figure 5. The latency of this reflex was 80 ms. The baseline threshold of RIII reflex at week 0 showed no significant difference between the coffee and juice groups. At week 2, the difference in threshold was still not significant. Similar results were observed for the values at week 4 (*P* = 0.805; Supplementary Table 2). The baseline EMG integral of RIII reflex induced by 1.2 Tr at weeks 0, 2, and 4 had no significant difference between the two groups (Supplementary Table 2).

The difference in the EMG integral was calculated as the integral change in pre-EA minus post-EA application. Repeated measurements failed to show a significant group-by-time point interaction at weeks 0, 2, and 4. This indicated that caffeine intake did not influence the effect of EA on RIII reflex (*P* > 0.05, Table 2).

## Discussion

The effect of caffeine on acupuncture analgesia in humans is yet to be clarified. In this study, the difference in PPT and PPTo of BL25 and BL57 after and before EA was significantly lower in the coffee group than in the juice group. The NRS score obtained during the experimental heat pain paradigm post- and pre-EA showed no significant difference after coffee intake for 4 weeks compared with that of juice intake. The changes in EMG integral from baseline to after EA were also not significantly different between the coffee and juice groups.

PPT, PPTo, and HPT are the main measurement indices of the threshold that primarily reflects the state of the peripheral nervous system (32). The results of the current study confirmed that the PPT and PPTo of BL25 and BL57 are increased by tolerance intensities of EA at ST36 and GB34. Many clinical trials have recently shown that static QST parameters, such as PPT, PPTo, and HPT, could be markedly elevated after needling (26, 33). The spinal innervation of the points detected for PPT, PPTo (BL25: L2, 3; BL57: S1, 2), and HPT (SP6: L3, 4) are of the same or adjacent to those stimulated in EA (ST36 and GB34: L4, 5). This effect suggests a strong involvement of segmental inhibition through A-fiber signaling (26). “Tolerance intensity” was verbally described as “high to tolerable but sub-noxious” and higher than “strong but comfortable,” and there were acupuncture sensations of fullness, heaviness, dull aching, or warmth (34, 35). The acupuncture sensation also suggests that Aδ and, possibly, C afferent fibers are activated. Stimulation of these fibers can elicit pain-modulating actions at the level of the dorsal horn as well as the release of endogenous opioids in the central nervous system, serotoninergic descending pain pathways, and diffuse noxious inhibitory controls (36–38).

The HPT is an index that reflects heat sensitivity. Most previous studies have indicated that HPT can be alleviated by acupuncture (32, 39) or adenosine (39, 40) in humans. However, some conflicting results have also been reported (41). In the current study, HPT was not significantly increased after EA, whereas PPT and PPTo were significantly increased. This may be explained as follows: First, the HPT is influenced by many factors (i.e., thermode area, ramp rate, anatomical site, glabrous or non-glabrous skin, and skin temperature) (42). Second, a systematic review and meta-analysis showed that 80% of the measured PPT were increased after acupuncture, while thermal detection findings were heterogeneous. Further, the assessments of pressure pain sensitivity may be more reliable than the assessment of heat pain sensitivity as the deeper tissues detected by PPT and PPTo may play an important role in many musculoskeletal pain conditions (33). One study reported a large variation in the statistical methods used when discussing the test-retest variability of the thermal threshold (43, 44). Conversely, good reproducibility of the mechanical thresholds has been reported (45).

The daily diet of the subjects was maintained, except that the coffee group consumed an extra 100–120 mg/day of caffeine, while the juice group did not consume extra caffeine. Low doses of caffeine exert an adjuvant anti-nociceptive effect in formulations containing aspirin, acetaminophen, and other non-steroidal anti-inflammatory drugs (NSAIDs) by inhibiting A_2a_AR and A_2b_AR (16, 46). Blockade of A1 receptors which occurs with moderate doses of caffeine can inhibit the anti-nociceptive effects of analgesics (47, 48). This effect on A1 receptors may lead to a decreased analgesic effect of acupuncture and transcutaneous electrical stimulation (49, 50), consistent with current study findings.

After 4 weeks of coffee consumption, PPT and PPTo were not significantly increased by acupuncture in the coffee group. Furthermore, the difference in PPT and PPTo values at week 4 were smaller in the coffee group than in the juice group. The different interventions between the two groups were as coffee intake in the coffee group contained moderate (100–120 mg/day) caffeine compared with the juice group. This suggests that moderate caffeine intake attenuates acupuncture efficacy on PPT and PPTo through the blockade of adenosine receptors, which play a vital role in the analgesic effect of acupuncture. One study reported that adenosine is released during acupuncture in mice and its anti-nociceptive actions required A_1_AR expression. Adenosine is degraded from ATP by several ectonucleotidases before the reuptake of ATP. Then, adenosine acts as an analgesic agent that suppresses pain through Gi-coupled A1-adenosine receptors. Adenosine was reuptaken by nucleoside transporters and degraded to inosine. The rapid clearance of adenosine in the extracellular space may shorten the anti-nociceptive effect of acupuncture (19, 50).

Consequently, we believe that acupuncture analgesia in humans is reversed after daily moderate caffeine intake (101–200 mg), consistent with previous results in animals (50). In North America, daily caffeine consumption ranges from 168 to 220 mg/day which is considered a high dose (51). Thus, our findings could provide some evidence to explain the different findings of acupuncture clinical trials between China and Western countries. A brief report showed that daily caffeine consumption did not influence the elevated PPT and HPT by acupuncture in healthy individuals (24). However, although, PPT and HPT were measured near the acupoints, they used manual acupuncture with a Deqi sensation, and the subjects were healthy individuals with low and high caffeine intake. Meanwhile, we used EA stimulation and the coffee group involved subjects with no habitual coffee habits and were given moderate doses of caffeine for 4 weeks. Thus, the heterogeneity between the two studies may be caused by differences in acupuncture stimulation and subjects (with or without coffee intake habits).

Changes in pain modulation processes, as reflected by dynamic psychophysical tests, are now being increasingly recognized as clinically relevant. The inhibition of experimental pain is tested at the bedside using the conditioned pain modulation (CPM) protocol, wherein the administration of two simultaneous painful stimuli typically results in pain inhibition. It is well-known that thermal, mechanical, or electrical pain stimuli used as the conditioning stimuli can inhibit a test stimulus. The current study showed that the changes from T1 to T3 were increased by EA intervention, and no group-by-time-point interaction was observed between the two groups at weeks 0, 2, and 4 (Supplementary Table 3).

CPM involves mechanisms at several levels, including modulation of the cerebral level *via* cortico-cortical interactions as well as modulation of the cerebrospinal level *via* descending pathways (52). Cerebral modulation has been shown to play a crucial role in the cognitive modulation of CPM through changes in attention, expectation, and emotion. In addition, modulation at the cerebral level plays a crucial role (53, 54). It is possible that caffeine cannot reduce the effects of acupuncture on the CPM NRS score because of the greater involvement of the central mechanisms in CPM modulation (55).

The nociceptive flexion reflex (NFR), composed of the RII and RIII reflexes, is widely used in pain research to investigate spinal and supraspinal influences on nociceptive processing in individuals with and without pain disorders. The European Federation of Neurological Societies guidelines stipulate that the RIII reflex is the most reliable nociceptive reflex for assessing treatment efficacy (56). Based on the observed EMG of the biceps femoris muscle response, the stimulation intensity required to elicit RIII is used as an objective index of the nociceptive threshold (56, 57). It is a polysynaptic and multi-segmental spinal reflex elicited by stimulation, which mainly activates nociceptive A-delta afferents.

In our previous study, transcutaneous EA of low intensities (below thresholds of RIII) on ST36 for 1 min reduced EMG induced by the RIII reflex in the ipsilateral leg. Activation of the endogenous opioid system could explain the “post-stimulus analgesic” effect (59). Moreover, some experimental evidence has revealed that neurotransmitters [e.g., serotonin (5-HT), dopamine, norepinephrine, gamma-aminobutyric acid, and glutamate] released by afferent fibers, descending terminations, or local interneurons in the dorsal horn modulate NFR in an inhibitory or excitatory manner (57). However, there are no reports on the relationship between neurotransmitters and caffeine. In this study, the RIII reflex also decreased from T0 to T6 by tolerant intensities of EA application for 20 min not only at week 0 but also at weeks 2 and 4. This supports that caffeine is not involved in the spinal nociceptive processing pathway and influences the effect of acupuncture on the RIII reflex. This could be because the targets for caffeine mainly focus on the peripheral adenosine receptor.

## Limitations

This research provides evidence on the possible causes for the difference in the effect of acupuncture between Western and Eastern populations who consume caffeine more or less differently. However, this study also has some limitations. First, the effects of the study setting cannot be completely ruled out, although experiments were conducted in a calm environment with a stable room temperature, and examiners were intensively trained and blinded to group allocation. As a psychophysical measure, QST may be influenced by environmental factors and examiner (58). In addition, the placebo effect of acupuncture was not discussed in this research. The effects of subjective expectation could not be ruled out because the participants were not blinded to their drink intervention. Second, the study population was mainly medical students. They may have had previous experience with psychophysical experiments that may have influenced our results. Third, the comparison was performed for only 4 weeks. There may be some differences between long-term active caffeine consumption for years and short-term experimental caffeine intake for weeks. Further studies on patients with chronic pain are needed to validate our findings.

## Conclusion

EA induces clinically relevant changes in the PPT, PPTo, CPM, and RIII reflexes in healthy adults. The effect of EA on PPT and PPTo was attenuated after caffeine intake, indicating a crucial role of adenosine mechanisms. This may also partially explain the difference in the analgesic effect of acupuncture between individuals who consume and do not consume caffeine.

## Data Availability Statement

The raw data supporting the conclusions of this article will be made available by the authors, without undue reservation.

## Ethics Statement

The studies involving human participants were reviewed and approved by China Ethics Committee of Registering Clinical Trials. The participants provided their written informed consent to participate in this study.

## Author Contributions

XG and BZ designed the study. KL performed QST measurements. KL and XC drafted the manuscript and prepared the figures and tables. MZhi coordinated trial conductance and performed data analysis. MZha performed part of the dynamic QST measurements. TZ performed acupuncture intervention. All authors have contributed substantially to both the research and the manuscript and read and approved the manuscript.

## Funding

The funding from the Scientific and Technological Innovation Project of the China Academy of Chinese Medical Sciences: No. CI2021A03402 and the projects of National Natural Sciences Foundation of China: Nos. 81473778, 81873389, and 81303054 contributed to XG, BZ, and KL supporting this study.

## Conflict of Interest

The authors declare that the research was conducted in the absence of any commercial or financial relationships that could be construed as a potential conflict of interest.

## Publisher's Note

All claims expressed in this article are solely those of the authors and do not necessarily represent those of their affiliated organizations, or those of the publisher, the editors and the reviewers. Any product that may be evaluated in this article, or claim that may be made by its manufacturer, is not guaranteed or endorsed by the publisher.
