# Supplementary material for: Caffeine Attenuates Electroacupuncture Effect on Pressure Pain Threshold and Tolerance in Healthy Individuals: A Randomized Controlled Trial
Source: Front Neurol. 2022 Jul 7;13:859624. doi: 10.3389/fneur.2022.859624 (PMC9301193; doi:10.3389/fneur.2022.859624)
Supplement: Supplementary file 1 [file Data_Sheet_1.DOCX]

| **Supplementary table 1 Group characteristics** | | | |
| --- | --- | --- | --- |
|  | **Coffee**  **(n=20)** | **Juice**  **(n=20)** | ***P*** |
| Age, y^*^ |  |  |  |
| Mean (SD) | 27.40 (5.06) | 25.35 (3.15) | 0.132 |
| Min-max | 24.0-32.0 | 21.0-37.0 |  |
| Sex, n (%)† |  |  |  |
| Male | 8 (40) | 11 (55) | 0.342 |
| Female | 12 (60) | 9 (45) |  |
| Expectancy scores^*^ | 7.80(0.29) | 7.77(0.66) | 0.972 |

^*^, independent t test.

†, Chi-square test.

| **Supplementary Table 2 Baseline static and dynamic QST values** | | | | |
| --- | --- | --- | --- | --- |
| **Parameters** | **Coffee** | | **Juice** | ***P*^*^** |
| **PPT (KPa)** |  | |  |  |
| BL25 Mean (SD) | | 616.07 (181.15) | 624.58 (272.84) | 0.908 |
| Min-max | 361.33-1081.00 | | 312.00-1334.00 |  |
| BL57 Mean (SD) | 443.70 (150.28) | | 435.40 (165.66) | 0.870 |
| Min-max | 263.00-750.67 | | 218.67-732.33 |  |
| **PPTo（KPa）** |  | |  |  |
| BL25 Mean (SD) | 911.25 (204.88) | | 911.14 (322.50) | 0.999 |
| Min-max | 591.00-1360.00 | | 411.67-1360.00 |  |
| BL57 Mean (SD) | 663.10 (217.17) | | 616.90 (219.09) | 0.508 |
| Min-max | 360.00-1243.67 | | 308.33-1289.00 |  |
| **HPT****(℃)** |  | |  |  |
| Mean (SD) | 46.06 (2.31) | | 46.31 (2.26) | 0.730 |
| Min-max | 42.00-49.73 | | 42.90-49.93 |  |
| **CPM NRS** |  | |  |  |
| 0wk Mean (SD) | 7.40 (1.58) | | 7.65 (1.51) | 0.775 |
| Min-max | 5.0-10.0 | | 5.0-8.0 |  |
| 2wk Mean (SD) | 7.30 (0.67) | | 7.65 (1.16) | 0.419 |
| Min-max | 6.0-8.0 | | 6.0-10.0 |  |
| 4wk Mean (SD) | 6.70 (1.06) | | 7.11 (2.14) | 0.593 |
| Min-max | 5.0-8.0 | | 3.0-10.0 |  |
| **RⅢ reflex threshold (mA）** | | | | |
| 0wk Mean (SD) | 3.43 (2.05) | | 3.34 (2.04) | 0.898 |
| Min-max | 1.50-7.50 | | 1.50-9.00 |  |
| 2wk Mean (SD) | 3.56 (1.99) | | 3.60 (1.36) | 0.940 |
| Min-max | 1.50-8.00 | | 1.50-6.74 |  |
| 4wk Mean (SD) | 4.39 (2.34) | | 4.22 (1.70) | 0.805 |
| Min-max | 2.00-10.35 | | 2.00-7.35 |  |
| **RⅢ reflex EMG integral (μV.s）** | | | | |
| 0wk Mean (SD) | 0.36 (0.26) | | 0.27 (0.09) | 0.140 |
| Min-max | 0.13-1.15 | | 0.11-0.47 |  |
| 2wk Mean (SD) | 0.36 (0.29) | | 0.41 (0.32) | 0.617 |
| Min-max | 0.11-1.19 | | 0.11-1.37 |  |
| 4wk Mean (SD) | 0.34 (0.22) | | 0.35 (0.12) | 0.906 |
| Min-max | 0.10-1.14 | | 0.15-0.55 |  |

^*^, independent t test.

PPT, pressure pain threshold; PPTo, pressure pain tolerance; HPT, heat pain threshold;

CPM, conditioned pain modulation; NRS, numerical rating scores, numerical rating scale 0-10, with 0 indicating no pain and 10 indicating severe pain.

| **Supplementary Table 3 Dynamic QST change** | | | | | | | | |
| --- | --- | --- | --- | --- | --- | --- | --- | --- |
| **Least square mean (SD)** | | | | | | | | |
|  | **0 Week†** | |  | **2 Week†** | |  | **4 Week†** | |
|  | **Coffee** | **Juice** |  | **Coffee** | **Juice** |  | **Coffee** | **Juice** |
| **CPM NRS** | | | | | | | | |
| T1 | 1.00(0.64) | -0.30(0.64) |  | 0.95(0.44) | 0.75(0.44) |  | 1.35(0.84) | 0.55(0.84) |
| T2 | 1.90(0.64) | 1.20(0.64) |  | 1.75 (0.44) | 1.37(0.44) |  | 1.65(0.84) | 1.76(0.84) |
| T3 | 2.45(0.64) | 2.00(0.64) |  | 2.25(0.44) | 1.90(0.44) |  | 2.20(0.84) | 2.39(0.84) |
| **RⅢ reflex EMG integral** | | | | | | | | |
| T1 | 0.05(0.03) | 0.01(0.04) |  | 0.05(0.06) | 0.08(0.05) |  | 0.02(0.03) | 0.07(0.02) |
| T2 | 0.08(0.03) | 0.02(0.04) |  | 0.01(0.06) | 0.09(0.05) |  | 0.01(0.03) | 0.07(0.02) |
| T3 | 0.12(0.04) | 0.03(0.03) |  | 0.01(0.06) | 0.13(0.05) |  | 0.01(0.03) | 0.09(0.02) |
| T4 | 0.09(0.03) | 0.02(0.03) |  | -0.01(0.06) | 0.16(0.05) |  | 0.03(0.03) | 0.06(0.02) |
| T5 | 0.08(0.03) | 0.03(0.03) |  | 0.02(0.06) | 0.14(0.05) |  | 0.04(0.03) | 0.07(0.02) |
| T6 | 0.00(0.03) | 0.02(0.03) |  | 0.04(0.06) | 0.11(0.05) |  | 0.02(0.03) | 0.04(0.02) |

†, General linear model for repeated measures with group as fixed effect, time point as random effect.

T1, T2, T3 following CPM NRS indicate during (30s), immediately after and 1 minutes after electrical acupuncture (EA), respectively.

T1-T6 following RⅢ reflex EMG integral indicate immediately after and 1 to 5 minutes after electrical acupuncture (EA), respectively.
